# Effects of antioxidant supplementation on oxidative stress balance in young footballers- a randomized double-blind trial

**DOI:** 10.1186/s12970-021-00447-z

**Published:** 2021-06-07

**Authors:** Błażej Stankiewicz, Mirosława Cieślicka, Sławomir Kujawski, Elżbieta Piskorska, Tomasz Kowalik, Justyna Korycka, Anna Skarpańska-Stejnborn

**Affiliations:** 1 Institute of Physical Education, Kazimierz Wielki University in Bydgoszcz, 2 Sportowa Str., 85-091 Bydgoszcz, Poland; 2 Department of Physiology, Collegium Medicum in Bydgoszcz, Nicolaus Copernicus University in Toruń, 24 Karłowicza Str, 85-092 Bydgoszcz, Poland; 3 Department of Hygiene, Epidemiology, Ergonomy and Postgraduate Education, Ludwik Rydygier Collegium Medicum in Bydgoszcz, Nicolaus Copernicus University in Toruń, 9 M. Curie Skłodowskiej Str., 85-094 Bydgoszcz, Poland; 4 Department of Pathobiochemistry and Clinical Chemistry, Collegium Medicum in Bydgoszcz, Nicolaus Copernicus University in Toruń, 9 M. Curie Skłodowskiej Str., 85-094 Bydgoszcz, Poland; 5 Institute of Food Sciences and Agrotechnics, University of Zielona Góra, Off-Campus Faculty in Sulechów, Pałac Kalsk - Kalsk 67, 66-100 Sulechów, Poland; 6 Department of Morphological and Health Sciences, Faculty of Physical Culture in Gorzów Wielkopolski, 13 Estkowskiego Str, 66-400 Gorzów Wielkopolski, Poland

**Keywords:** Athletes, Total antioxidant capacity, Radical oxygen species, Exercise, Diet supplement, Football players

## Abstract

**Background:**

Intensive physical exercise that competitive sports athletes participate in can negatively affect their pro-oxidative–antioxidant balance. Compounds with high antioxidant potential, such as those present in chokeberry (*Aronia melanocarpa*), can prevent these adverse changes. We here investigated the effect of antioxidant supplementation on oxidative stress balance in young footballers.

**Methods:**

The study was designed as a double-blind randomized trial. Diet of a group of young football players (male; *n* = 20; mean age, 15.8 years-old) was supplemented with 200 ml of chokeberry juice per day, for 7 weeks. The players were randomly assigned to the experimental (supplemented, FP-S; *n* = 12) and control (placebo, FB-C; *n* = 8) groups. Before and after the supplementation period, the participants performed a beep test. Venous blood was sampled for serum analysis before, immediately after, 3 h, and 24 h after the beep test. Serum levels of thiobarbituric acid reactive products, 8-hydroxy-2′-deoxyguanosine, total antioxidant capacity, iron, hepcidin, ferritin, myoglobin, and albumin, and morphological blood parameters (red blood cells, (RBC), haemoglobin (HGB), haematocrit (HCT) mean corpuscular volume (MCV) mean corpuscular haemoglobin (MCH), mean corpuscular haemoglobin concentration (MCHC), and lactic acid) were determined.

**Results:**

Chokeberry juice supplementation did not significantly affect the outcome of the beep test. The supplementation did not significantly affect any of the morphological, biochemical, or performance parameters analysed.

**Conclusions:**

Chokeberry juice supplementation did not affect the measured parameters in the studied population, which may indicate insufficient antioxidant capacity of the juice.

## Background

Increased metabolic activity during physical exercise is accompanied by an increased generation of reactive oxygen species, which may lead to disorders of the immune system function [1, 2]. This applies primarily to high-intensity and long lasting exercise. The underlying mechanism is not fully understood [3].

It is believed that excessive production of free oxygen radicals leads to multiple changes in the body metabolism [4]. The observed rapid increase in oxygen radical production leads to erythrocyte damage, as a consequence of lipid peroxidation, which increases their sensitivity to degradation [5]. Increased haemolysis, in turn, leads to a substantial increase in the concentration of redox-active free iron in the blood [6]. Circulating free iron may be toxic and destructive to cell components and body fluids. Upon oxidative stress, activation of the immune system and inflammation are also observed, as an early defence response of the body. Most likely, oxidative stress is ‘sustained’ in this manner also during post-workout recovery [7].

Peaks of alternating oxidative stress markers in the blood after a bout of physical exercise involving concentric and eccentric contractions are observed between 0 and 4 h, and 48 and 96 h after the exercise bout, respectively [8, 9]. Increased ionized iron levels in the blood can contribute to the intensification of free radical reactions [10], which weakens the immune system, thereby increasing susceptibility to infection [11–13]. Acute post-exercise depression of the immune system may result not only in an increased frequency of infection among sports competitors, but also in an increased percentage of cases (especially of upper respiratory tract infections) and a prolonged duration of infection. Furthermore, in many situations, depression of the immune system may lead to an increased possibility of injury and hinders tissue regeneration [14].

Chokeberry (*Aronia melanocarpa*) contains a wide range of biologically active compounds, including polyphenols, such as anthocyanins, flavonoids, and phenolic acids [15, 16]. There is evidence that compounds present in chokeberry positively affect the immune system and oxidative balance [17]. That is particularly attributed to anthocyanins, which are present in copious amounts in the chokeberry fruit [18]. These compounds can impact the immune system [19, 20], reduce oxidative stress, and chelate iron ions [21]. Anthocyanin supplementation might reduce post-exercise muscle soreness [22] and improve performance parameters [23].

Analysis of data from athlete studies available in various scientific databases, as well as numerous scientific reports on non-training individuals and animals, suggests that the endogenous defences against oxidative stress of an organism subjected to an intense exercise load are insufficient [24]. Further, dietary preparations rich in anthocyanins may be an important factor alleviating the adverse effects of extreme exercise loads. It therefore may be advisable for the competitors’ diet to contain plants rich in anthocyanins, which not only have the ability to form stable complexes with the transition metals but also increase the body’s antioxidant potential. Such supplementation can reduce oxidative stress, greatly reducing post-exercise inflammatory processes, and contribute to an increase in ergogenic potential [24].

The aim of the current study was to analyse the effect of 7-week supplementation with chokeberry juice on pro-oxidative–antioxidant balance parameters and selected iron level parameters in professional young footballers during football season, compared with a placebo group in a randomized double-blind trial.

## Methods

### Participants

The study was designed as a double-blind randomised controlled trial with parallel groups. After screening with respect to the inclusion and exclusion criteria by laboratory assistants, 20 young male semi-professional footballers (15.8 ± 0.7-years-old) from Międzyszkolny Uczniowski Klub Sportowy (MUKS) Zawisza Bydgoszcz club (Bydgoszcz, Poland), participating in the Central Junior League competitions, took part in the study. The subjects were randomly assigned to the supplemented (*n* = 12; FP-S) or the placebo group (*n* = 8; FP-C). Each group was similar in terms of the anthropometric data and the position on the football field (Table 1). Basic characteristics of the study group are summarized in Table 1. The participants receiving chokeberry juice or a placebo followed a uniform training load scheme. Training loads for the entire experimental period (microcycle) are shown in Table 2. Load time intensity from the beginning of the season until the end of the experiment is summarized in Table 3.

**Table 1:** Characteristics of the examined group

*N* = 20	Age [years]	Years of training [years]	Height [cm]	Weight [kg]	BMI [kg/m2]	Body fat [%]
$\overline{x}$	15.8	6.5	182.9	72.4	21.6	13.2
Min.	15.2	5	168	61.7	18.8	10.6
Max.	16.8	8	190	80.8	24.1	16.8
σ	0.7	0.8	5	5.6	1.3	1.8
V	4	13.6	2.7	7.7	6.1	13.3

*N* population, *cm* centimetre, *kg* kilogram, *%* percent, − arithmetic average, *min*. minimum value, *max.* maximum value, *σ* standard deviation, *V* coefficient of variation

**Table 2:** Training loads of the whole experimental time

Training	Monday	Tuesday	Wednesday	Thursday	Friday	Saturday	Sunday
Training total time	45–60 min	90 min	90 min	90 min	45–60 min	120 min	30 min
Time of a single exercise	Short 2–4 min	Medium 4–8 min	Long 8–20 min	Very short 1–2 min	Short 2–5 min	Long 45 min	Long 30 min
Training loads, scale 1–10*	1–2	4–6	5–8	2–4	3–5	8–10	1–2
Training content	Active regeneration. Large forms of tactics with technique breaks.	Elements of the game in attack and defence in a limited field of play.	Maximum intensity. Endurance. Small and large games.	Game speed without much resistance. Defence against counterattack.	Force-speed stimulation. Tactical games and exercises with an accent of speed.	Control/ championship match.	Active regeneration e.g.: jogging, walking, swimming.

*min* minutes, * training loads scale where 1 means training at the lowest intensity and 10 means training at the highest intensity

**Table 3:** Summary of microcycle intensity (from the beginning of the season till the end of experiment)

Work load characteristics	min.	%
Aerobic	2922	64,9
Mixed intensity	1322	29,4
Anaerobic lactate acid dependent	177	3,9
Anaerobic not lactic acid dependent	143	3,2
Totality	4499	100

All subjects were informed about the purpose of the research and the procedures, and voluntarily agreed to participate in the study. The research was conducted according to the Declaration of Helsinki and was approved by the local Bioethics Committee at Collegium Medicum in Bydgoszcz (approval no. KB 382/2017). All players were assessed with respect to the inclusion and exclusion criteria, and were asked not to use any supplements (vitamins, ergogenic supplements, herbal extracts, caffeine, theine, etc.) 2 weeks before and during the experiment. One week before the exercise test and during the experiment, the participants adopted similar eating habits. They were asked to eat balanced meals prepared based of the daily energy requirements in relation to age and physical activity. Substances that could interfere with the test results, containing large amounts of anthocyanins, phytosterols, and antioxidants were excluded from the meals. All meals were prepared according to the guidelines of professional sports nutrition by a sport nutritionist, as recommended by the Polish Football Association [25].

### Study design

**Fig. 1:**
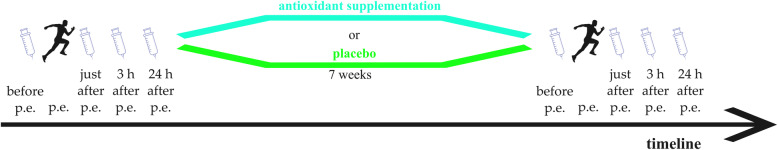
Research protocol. p.e., physical exercise test

The participants were randomly divided into two groups: the supplemented group (*n* = 12), which received 200 ml of chokeberry juice (100 ml twice a day, in the morning and in the evening) for 7 weeks; and the control group (*n* = 8), which received a placebo at the corresponding times, according to published guidelines [17]. In previous studies, the average duration of chokeberry supplementation tested was 6 to 8 weeks [26]. The research protocol scheme is presented in Fig. 1.

### Physical exercise program

During the entire experimental period, all subjects followed their regular physical exercise program. The physical exercise program was planned by the main coach of the team, and was the same for both groups. The training program microcycle (presented in Table 2) consisted of a uniform pattern of tasks performed during the game season, during which the research was conducted, with the intensity level of a given training unit expressed on a scale from 1 to 10 (the training loads scale).

### Supplementation

The anthocyanin content was determined to be 165.3 mg/100 ml of juice. Briefly, the anthocyanin pigment content was analysed by high-performance liquid chromatography, as described by Oszmański and Sapis [27]. For the analysis, LC Agilent Technologies 1200 Rapid Resolution (Waldbronn, Germany) system equipped with a UV–Vis detector (DAD 1260, Waldbronn, Germany) and Zorbax SB-C18 column (4.6 × 150 mm, 5 μm) (Agilent, Wilmington, Delaware, USA) were used. Separation was achieved using a reversed-phase system with gradient elution. Chromatographic conditions were as follows: injection volume, 20 μm; flow rate, 1.0 ml/min; solvent A, 10% formic acid in water; solvent B, 10% formic acid, 30% acetonitrile, 60% water. The following gradient was used: 0–8 min 20–40% B, 8–15 min 40–50% B, 15–16 min 50–100% B, 16–20 min 100% B (isocratic), 20–23 min 100–20% B. Chromatographic data were acquired at 400 to 600 nm, and integrated at 520 nm for anthocyanins. The results are expressed as cyanidin-3-*O*-glucoside (external standard) (LGC Standards, Bury, UK) (mg/100 g or %). Cyanidin-3-*O*-glucoside was dissolved in water, and the chokeberry juice was diluted 10 times in redistilled water and filtered through 0.45-μm filter prior to analysis.

Subjects in the control group were given the placebo containing 6.6% solution of betaine [(CH_3_^+^)_3_ N^+^ · CH_2_ COO^−^] and 1% solution of citric acid. The placebo was identical in appearance and taste to chokeberry juice, and both were given in 200-ml numbered sintered glass bottles. The label codes were decoded after the examination of all biochemical factors after intervention completion. The participant play position or volume of competition play (starters vs. non-starters) was not considered in the randomization. Both the chokeberry juice and placebo were produced by MLB Biotrade Sp. z o.o., Poland (Poznan, Poland). The players and researchers were blinded to group assignment.

### Antioxidant capacity of chokeberry juice

The antioxidant capacity of chokeberry juice was determined using 2,20-azinobis (3-ethylbenzthiazoline-6-sulfonic acid) (ABTS) and 2,2-di-phenyl-1-picrylhydrazyl radical (DPPH) methods at the Lubuskie Centre for Innovation and Agricultural Implementation of the University of Zielona Góra (Sulechów, Poland). ABTS, DPPH, and other reagents were purchased from Sigma Aldrich (St. Louis, MO, USA). The juice contained 8.83 mg/ml ABTS and 7.62 mg/ml DPPH.

### Physical exercise test

Before and after the 7-week supplementation period, all players performed the maximal multistage 20-m shuttle run test (the ‘beep test’) [28]. The test was carried out in a full-size sports hall with a classic surface, from 9:30 AM to 10:30 AM. The participants were asked to eat a light meal approximately 2 h prior to the test. They were instructed not to consume alcohol, caffeine, theine, or taurine on the test day. VO_2max_ was calculated indirectly based on the results of the physical exercise test, as described elsewhere [29], on the assumption that retroextrapolated VO_2max_ is not substantially different from VO_2max_ measured directly [29]. The physical exercise test took place on a Tuesday instead of the planned training session. The supplementation began on the following Monday and ended after 7 weeks on a Sunday. After the supplementation period, the test was repeated on the following Tuesday instead of the planned training session. During the test, the air temperature was 19.1 °C and humidity was 51%. All the tested players were informed about the test procedures and were additionally motivated by the trainer to make maximum effort.

### Blood sampling and analysis

Blood samples were taken for analysis at four time points at the beginning and at the end of the supplementation period: before, immediately after, and 3 and 24 h after the beep test. These time points were selected because the levels of hepcidin and related parameters (interleukin, IL, 6) achieve a maximum 3 h after exercise [30, 31]. Further, blood sampling after 24 h allows determination whether the tested parameters have returned to the resting values. That is important because the training program consisted of daily physical exercise sessions in the examined subjects. Blood for serum analysis was collected from the ulnar vein into 9-ml serum tubes containing a coagulant (Sarstedt, Germany). The blood was centrifuged (3000 rpm, 10 min), and the serum was aliquoted, frozen in liquid nitrogen, and stored at − 80 °C until analysis.

To determine the morphological blood parameters (red blood cells, RBC; haemoglobin, HGB; haematocrit, HCT; mean corpuscular volume, MCV; mean corpuscular haemoglobin, MCH; and mean corpuscular haemoglobin concentration, MCHC), venous blood was collected into 5-ml tubes containing EDTAK_2_ as the anticoagulant. Morphological examinations were performed using flow cytometry on Sysmex XS-1000i apparatus (Kobe, Japan).

Iron levels were determined in plasma taken from lithium heparin and determined by in vitro IRON 2 test for the quantitative determination of iron in human serum and plasma, using Roche/Hitachi Cobas c. system and a Cobas c 501 analyser (Cobas, Rotkreuz, Switzerland).

Lactic acid (LA) levels were measured in capillary blood collected from the earlobe before and immediately after the beep test, using a Dr. Lange Plus LP20 biochemical analyser (Dr. Lange, Berlin, Germany).

For detailed analysis of changes in the body’s iron management, total antioxidant levels, and the inflammatory cell response, the following enzyme-linked immunosorbent assay (ELISA) kits were used, according to the manufacturers’ instructions: ferritin ELISA kit EIA-1872, IL-6 ELISA kit EIA-4640, myoglobin ELISA kit EIA-3955, and hepcidin 25 (bioactive) HS ELISA kit EIA-5782, from DRG International, Inc. (Springfield, New Jersey USA); human thiobarbituric acid reactive substances (TBARS) ELISA kit (catalogue no. 201–12-7298) and human 8-oxo-2′-deoxyguanosine (8-OHdG) ELISA kit (catalogue no. 201–12-1437), from Shanghai SunRed Biological Technology Co. Ltd. (Shanghai, China); human albumin ELISA kit (catalogue no. EA2201–1) from Assaypro LLC (St. Charles, MO, USA); and TAC Fast Track DM P-4100 from LDN Labor Diagnostika Nord GmbH & Co. KG (Nordhorn, Germany). Thermo Scientific Multiscan GO microplate spectrophotometer produced by Fisher Scientific Finland (Vantaa, Finland) was used for the analyses.

### Statistical analysis

Sample size calculation was done based on previous results on the effects of chokeberry supplementation on TBARS levels in males [32], as the variable of primary interest in the study, using a calculator available online [https://powerandsamplesize.com/Calculators/Compare-2-Means/2-Sample-1-Sided]. As in the previous study [32], sample size was increased in the intervention group by setting the sampling ratio as 1.5. The power was set to 0.8, with the type I error rate of 5%. The calculated sample size in the intervention group was *n* = 12. Shapiro–Wilk W test and visual histogram assessment were used to test the assumption of normality.

Two-factor analysis of variance (ANOVA) with group coefficient (supplemented group/placebo group) and time (before/after supplementation) was selected for the analysis of physical fitness variables using aligned rank transform for nonparametric factorial ANOVA with ARTool package for R [33]. Post-hoc test for differences of differences was done using the R package phia [34]. Partial *eta*-squared was calculated to assess the effect size of interaction in two-way ANOVA. To assess the dynamics of biochemical parameters in response to the physical exercise test, a linear mixed model fit by REML with *t*-tests using Satterthwaite’s method was implemented in the R statistical packages lme4 and lmerTest [35, 36]. Subject factor was set as a random effect. Time (before vs. just after vs. 3 h after vs. 24 h after the physical exercise test in the case of biochemical parameters; and before vs. 3 h after the physical exercise test in the case of blood morphometry parameters), group (placebo vs. supplemented), and intervention (before vs. after the physical exercise programme) were set as fixed effects. Interaction between fixed effects and the confidence interval (CI, 95%) for determining the interaction were calculated. Mean values and standard deviation (SD) are reported. Alpha level was set to 0.05.

## Results

There was no significant interaction of time × group and VO_2max_ (58.82 ml/kg/min before vs. 60.35 ml/kg/min after in the juice group, 58.48 ml/kg/min before vs. 60.36 ml/kg/min after in the placebo group) (F = 0.04, *p* = 0.84, partial *eta*-squared = 0.002). Likewise, there was no significant interaction of time × group and the distance covered in the beep test (2528.33 (222.9) m, level 13, interval 8 before supplementation vs. 2631.67 (222.1) m, level 13, interval 13; after supplementation in the juice group: 2450 (384.9), level 13, interval 7 before supplementation vs. 2610 (228) m, level 13, interval 13 after in the placebo group; F = 0.02, *p* = 0.9, partial *eta*-squared = 0.001).

Interaction between the intervention × group was noted for albumin levels (*p* = 0.03). However, the albumin levels were not significantly affected by the physical exercise test, intervention, and group (Table 4).

**Table 4:** The impact of chokeberry supplementation on selected parameters of inflammation and iron management

Parameters	Supplemented group		Placebo group		*p*-value interaction intervention * effects of physical exercise test * group
	before supplementation Mean (SD)	after supplementation Mean (SD)	before supplementation Mean (SD)	after supplementation Mean (SD)	
**Albumin [μg/ml]**					
Before	4.55 (1.1)	3.55 (0.7)	4.65 (0.9)	4.02 (1.0)	0.23
After	4.85 (0.6)	3.79 (0.6)	5.34 (1.7)	3.69 (0.6)	
3 h after	4.46 (0.8)	3.31 (0.5)	5.57 (1.8)	3.63 (0.8)	
24 h after	4.33 (1.2)	3.73 (0.9)	5.15 (1.8)	3.23 (0.4)	
**Myoglobin [ng/ml]**					
Before	15.23 (7.5)	14.11 (4.3)	19.35 (15.6)	17.64 (8.9)	0.91
After	17.50 (5.8)	17.77 (6.9)	21.21 (17.9)	22.82 (11.0)	
3 h after	19.17 (8.3)	14.98 (6.1)	24.16 (14.7)	24.18 (12.3)	
24 h after	28.85 (13.6)	17.60 (13.9)	28.25 (16.4)	16.30 (7.5)	
**IL-6 [pg/ml]**					
Before	47.44 (13.1)	48.42 (18.8)	42.80 (7.0)	44.51 (4.2)	0.99
After	49.97 (12.7)	54.25 (24.5)	43.83 (2.3)	49.11 (4.8)	
3 h after	51.35 (23.1)	47.47 (10.4)	45.75 (11.9)	45.33 (4.0)	
24 h after	46.84 (13.7)	46.98 (10.8)	43.97 (5.0)	45.18 (3.9)	
**Hepcidin [ng/ml]**					
Before	6.99 (3.5)	9.31 (12.9)	7.34 (8.6)	4.74 (1.6)	0.75
After	7.24 (4.3)	11.29 (16.5)	7.55 (8.8)	4.53 (2.2)	
3 h after	7.56 (4.9)	12.42 (15.4)	8.96 (9.1)	7.04 (4.1)	
24 h after	8.27 (5.6)	8.69 (11.5)	4.39 (2.8)	4.05 (1.4)	
**Ferritin [ng/ml]**					
Before	12.11 (7.2)	13.08 (8.6)	10.09 (4.8)	11.55 (4.3)	0.85
After	12.46 (8.3)	14.84 (9.5)	11.14 (5.7)	13.12 (5.9)	
3 h after	11.17 (6.2)	12.76 (8.8)	10.72 (6.0)	10.68 (4.3)	
24 h after	11.79 (7.2)	13.14 (9.3)	10.78 (5.4)	11.04 (6.8)	
**Iron [μg/ml]**					
Before	97.12 (19.8)	104.67 (43.3)	114.74 (32.5)	120.19 (23.8)	0.13
3 h after	78.35 (20.1)	81.37 (35.2)	88.71 (24.4)	125 (16.7)	

(*SD*) standard deviation, *μg/ml* micrograms/millilitre, *ng/ml* nanograms/millilitre, *pg/ml* picograms/millilitre, *before* before the test, *after* after the test, *3 h after* 3 h after the test, *24 h after* 24 h after the test

Hepcidin levels were not significantly affected by the physical exercise test, intervention, and group (Table 4). Interaction between the intervention and group was observed for iron levels (*p* = 0.0495). However, the iron levels were not significantly affected by the physical exercise test, intervention, and group (Table 4). Biochemical analysis of the remaining selected parameters of inflammation did not reveal any significant interactions in the supplemented or placebo groups (Table 4).

Further, TBARS levels, 8-OHdG levels, and other pro-oxidative–antioxidant balance indicators were not significantly affected by the physical exercise test, intervention, and group (Table 5). Chokeberry supplementation did not significantly affect blood morphology (Table 6). Finally, no significant changes in the body weight, body mass index (BMI), and adipose tissue were observed after supplementation in any group (Table 7).

**Table 5:** Influence of chokeberry supplementation on selected parameters of pro-oxidative-antioxidant balance

Parameters	Supplemented group		Placebo group		*p*-value interaction intervention * effects of physical exercise test * group
	before supplementation Mean (SD)	after supplementation Mean (SD)	before supplementation Mean (SD)	after supplementation Mean (SD)	
**TAC [mmol/l]**					
Before	1.36 (0.3)	1.94 (0.7)	1.03 (0.3)	1.76 (0.5)	0.49
After	0.97 (0.3)	0.74 (0.4)	0.81 (0.3)	0.97 (0.3)	
3 h after	1.18 (0.3)	1.52 (0.4)	1.00 (0.3)	1.45 (0.2)	
24 h after	1.13 (0.3)	1.77 (0.3)	1.25 (0.5)	1.75 (0.5)	
**TBARS [nmol/ml]**					
Before	22.45 (1.9)	17.11 (5.3)	23.92 (3.5)	14.69 (4.6)	0.96
After	22.26 (3.1)	18.58 (7.8)	23.37 (3.9)	14.36 (3.6)	
3 h after	21.01 (2.9)	18.53 (6.8)	24.09 (3.6)	16.18 (3.6)	
24 h after	21.47 (3.2)	16.85 (5.7)	24.24 (6.2)	15.18 (5.1)	
**8-OHdG [ng/ml]**					
Before	3.2 (0.8)	2.04 (0.5)	4.02 (1.4)	1.87 (0.3)	0.41
After	3.01 (0.8)	2.28 (0.7)	4.13 (1.6)	2.09 (0.4)	
3 h after	3.04 (0.9)	2.12 (0.5)	3.28 (1.0)	1.98 (0.3)	
24 h after	3.12 (0.6)	1.99 (0.8)	3.35 (1.0)	1.73 (0.2)	

*SD* standard deviation, *mmol/l* millimoles/litre, *nmol/ml* nanomoles/millilitre, *ng/ml* nanograms/millilitre, *TBARS* thiobarbituric acid reactive substances, *TAC* total antioxidant capacity, *8-OHdG* 8-oxo-2′-deoxyguanosine, *before* before the test, *after* after the test, *3 h after* 3 h after the test, *24 h after* 24 h after the test

**Table 6:** Effects of periods of antioxidant supplementation on blood morphology before and after physical exercise test

	Supplemented group		Placebo group		***p***-value interaction intervention * effects of physical exercise test * group
Parameters	before supplementation Mean (SD)	after supplementation Mean (SD)	before supplementation Mean (SD)	after supplementation Mean (SD)	
**WBC [K/μl]**					
before	7.80 (1.5)	7.05 (1.7)	7.57 (2.7)	6.79 (2.6)	0.78
3 h after	9.75 (2.2)	9.13 (1.5)	9.95 (4.2)	9.61 (3.0)	
**RBC [× 10**^**12**^**/l]**					
before	4.89 (0.2)	5.07 (0.3)	4.75 (0.2)	4.98 (0.3)	0.82
3 h after	4.86 (0.2)	4.87 (0.3)	4.68 (0.3)	4.77 (0.2)	
**Hb [g/dl]**					
before	14.45 (0.6)	14.86 (0.9)	14.21 (0.5)	14.93 (0.8)	0.86
3 h after	14.37 (0.5)	14.34 (0.9)	14.01 (0.7)	14.23 (0.8)	
**Hct [%]**					
before	40.76 (1.6)	42.48 (2.2)	39.70 (1.5)	42.03 (2.4)	0.79
3 h after	40.26 (1.5)	40.40 (2.1)	38.91 (2.3)	39.43 (2.5)	
**Fe [μg/dl]**					
before	97.12 (19.8)	104.67 (43.3)	114.74 (32.5)	120.19 (23.8)	0.13
3 h after	78.35 (20.1)	81.37 (35.2)	88.71 (24.4)	125 (16.7)	
**LA [mmol/l]**					
before	1.45 (0.3)	1.60 (0.3)	1.56 (0.3)	1.48 (0.2)	0.72
after	9.85 (2.4)	10.58 (1.8)	9.62 (1.9)	10.56 (1.8)	

*SD* standard deviation, *WBC* white blood cells, *RBC* red blood cells, *Hb* haemoglobin, *Hct* haematocrit, *Fe* iron, *LA* lactate acid

**Table 7:** Body mass, body mass index and body fat level changes

Parameters	Supplemented group		Placebo group		*p*-value intervention * group interaction
	before supplementation Mean (SD)	after supplementation Mean (SD)	before supplementation Mean (SD)	after supplementation Mean (SD)	
Weight [kg]	68.42 (6.7)	69.4 (6.4)	63.66 (5.6)	64.44 (5.5)	0.97
BMI [kg/m2]	21.1 (1.9)	22.1 (1.7)	20.51 (1.4)	20.75 (1.3)	0.96
Body fat [%]	12.9 (1.6)	10.8 (1.9)	13.8 (1.9)	11.7 (0.6)	0.84

*SD* standard deviation, *BMI* body mass index, *body fat* percentage body fat

## Discussion

Physical exercise that competitive sports athletes participate in may disturb body homeostasis, which in turn may lead to reduced sports performance and deterioration of health [37]. According to the available literature, compounds found in chokeberry have strong antioxidant activity [38]. Anthocyanins are key in this respect, as they prevent excessive formation of free radicals, namely, the superoxide, hydroxyl, nitrite, and chlorine radicals [39, 40]. The anti-radical activity of anthocyanins increases with the number of hydroxyl groups on the B ring and the arylation of sugar residues with phenolic acids. Van Acker et al. [41] showed that the ability of anthocyanins to remove nitric oxide radical (•NO) is 100 times higher than that of the endogenous antioxidant glutathione. Anthocyanins chelate transition metal ions (e.g. iron and copper) via of the presence of hydroxyl groups on the C ring [42]. Another important feature of anthocyanins from the health perspective is their ability to inhibit lipid peroxidation [43]. This property can be of great importance for reducing haemolysis induced by intense physical exertion [44, 45].

In the current study, the antioxidant potential of administered chokeberry juice was determined using two methods, DPPH and ABTS, as 8.83 mg/ml and 7.62 mg/ml (relative to the activity of the Trolox reference compound), respectively. This indicates that the antioxidant potential of the juice was relatively low compared with that of chokeberry extracts and fresh fruit [46]. This might explain the lack of statistically significant effects of chokeberry supplementation in the current study.

In the current study, the chokeberry juice had no effect on free radical damage, as determined by the measurements of TBARS and 8-OHdG levels (Table 5). Petrovic et al. [32] tested the effects of 4-week chokeberry juice supplementation (100 ml/d) in handball players. The supplementation resulted in small changes in the lipid profile and in reduced TBARS levels in blood; however, these changes were observed only in men. By contrast, Cikiriz et al. [47] tested the effects of 12-week chockeberry extract supplementation (30 ml/d) in another group of handball players. Before the supplementation, and after 6 and 12 weeks, the subjects performed maximal physical exercise on a treadmill. Some beneficial changes, namely, a reduction of TBARS level, and increase in haemoglobin content, erythrocyte counts, and high-density lipoprotein (HDL) levels, were reported after 6 weeks of supplementation. However, the composition of the supplement and its antioxidant potential were not described in that study [47].

Intriguingly, García-Flores et al. [48] tested a combination of chokeberry extract with citrus juice (200 ml of drink containing 95% fresh citrus juice and 5% chokeberry extract) in triathlon riders. This combination of ingredients significantly reduced post-exercise changes in the levels of DNA damage markers determined in the plasma and urine [48]. The above changes were observed with juice with the anthocyanin content of 53.4 mg. In the current study, the amount of anthocyanins was four times higher, i.e. 230.6 mg. Hence, it is likely that a combination of polyphenols, rather than the anthocyanin content, plays a role in reducing DNA damage.

Analysis of the available literature indicates that the advantage of compounds derived from chokeberry is their comprehensive effect on both the immune system and reduction of oxidative stress, including the ability to chelate iron ions, which seems to be a key element not only for iron management. For this reason, we expected it to reduce markers of oxidative stress. We therefore expected chokeberry juice supplementation to reduce oxidative stress markers. However, we observed a statistically insignificant reduction of the average values of oxidative stress markers tested after the second beep test (after supplementation) in both, the supplemented and control groups. This may reflect the players’ adaptation to the applied exercise load. Zügel et al. [49] analysed the cumulative effect of training stress in highly qualified athletes practising rowing, focusing on hepcidin and parameters related to iron management. The authors showed that the levels of hepcidin and ferritin, acute-phase proteins, were a sensitive indicator of changes in the training load (exercise volume and intensity). In the current study, football players were subjected to the same training load throughout the entire study period, which probably explains the lack of statistically significant differences in the levels of hepcidin and ferritin. In another study [50], the effect of physical exercise and supplementation with juice high in polyphenols (containing chokeberry extract, among other ingredients) on hepcidin levels was analysed in a group of triathletes of both sexes. No significant impact of the supplementation on hepcidin levels was noted; instead, hepcidin level reduction was shown to be associated with the adaptation of players’ bodies to the applied exercise load.

In the current study, iron levels 3 h after the beep test decreased in the supplemented group and increased in the control group; however, these differences were not statistically significant. Similar changes in iron levels after supplementation with chokeberry (150 ml/d) were observed in a group of rowers in our previous study [51]. According to a cell line-based study, anthocyanins are inserted into the outer part of the erythrocyte membrane [52]. Their presence in the hydrophilic part of the membrane forms a protective shield against free radicals, among others, thus rendering them safe and effective antioxidants. Probably this fact may be explained by the decreased level of iron compared to the control group [52].

Iron ion chelation by active compounds present in chokeberry [53] might counteract muscle fibre damage. However, in the current study, we did not observe significant changes in the levels of myoglobin, a marker of muscle fibre damage. Specifically, myoglobin levels showed a downward trend in the group supplemented with chokeberry, but increased in the control group 3 h after physical exercise test after supplementation.

Anthocyanins modulate inflammation, both because of their ability to sequester iron [54] and because of their regulation of various components of the immune system involved in the development of inflammation [55]. For instance, Ohgami et al. [56] showed that chokeberry extract has a strong anti-inflammatory effect on endotoxin-induced uveitis in rat. The authors also observed that the number of inflammatory cells, protein concentration, and levels of NO, pyrogenic prostaglandin E2, and tumour necrosis factor α in the aqueous humour in animal groups treated with crude chokeberry extract were significantly reduced, and the effect size was dose-dependent [56]. Consequently, standardization of the content of anthocyanin compounds, which play a key health-protective role, in chokeberry products should be considered for their use.

One of the potential limitations of the current study is the relatively small sample size (*n* = 12 and *n* = 8 in the supplemented and placebo groups, respectively). Future studies examining the antioxidant effect of chokeberry in professional athletes should incorporate larger sample size and/or implement crossover design. In addition, participant play position or volume of competition play (starters vs. non-starters) was not considered in the randomization process in the current study. These factors should be addressed in future studies. Furthermore, the diet regime was not controlled in the current study, and potential changes in the amount of fruit and/or vegetable consumption might have interfered with the intervention [57]. In addition, subject compliance was not controlled. Implementation of a web-based app with reminders of the supplementation time and dosage might potentially resolve this problem.

## Conclusions

Chokeberry juice supplementation of footballers’ diet did not affect the indicators of inflammation and iron management, pro-oxidative-antioxidant balance, and blood morphology determined during the applied stress test. This could be explained by both, good adaptation of the athletes to the applied exercise load and the insufficient antioxidant capacity of the chokeberry juice tested. Because of the relatively small sample size in the current study, further studies should be conducted with a larger sample and/or implementation of crossover design. Further research should consider the supply of chokeberry in a more concentrated form, e.g. as a concentrate or lyophilizate, to compare the effects of chokeberry supplement types (e.g. juice, concentrate, mixtures) or of various levels of antioxidant potential. Extremely intensive physical exercise can potentially lead to excessive muscle damage, which would decrease training progress. Hence, future research should examine the possible mitigating effects of chokeberry juice on muscle damage and training progress improvement.

## Data Availability

Data and publication materials are available from the corresponding author on reasonable request.

## Abbreviations

ANOVA: Analysis of variance
n: Sample size
F: Result of variance analysis
t: Ratio of the departure of the estimated value of a parameter from its hypothesized value to its standard error/result of Satterthwaite’s method
p: *P*-value
%: Percent
σ: Standard deviation
SD: Standard deviation
$$ \overline{x} $$: Arithmetic average
V: Coefficient of variation
min: Minimum
max: Maximum
R: Language and environment for statistical computing and graphics
phia: Post-hoc interaction analysis R package
ggplot2: Data visualization package for the statistical programming language R
REML: Restricted maximum likelihood
FDR: False discovery rate
CI: *Confidence interval*
DPPH: 2,2-diphenyl-1-picrylhydrazyl
ABTS: 2,2′-azino-bis (3-ethylbenzothiazoline-6-sulphonic acid)
UV-VIS: *Ultraviolet*–*visible*
OH: Hydroxyl radical
URTI: *Upper respiratory tract infection*
vs: Versus
mg: Milligram
ml: Millilitre
ng: Nanogram
pg: Picogram
μg: Microgram
°C: Degrees Celsius
VO2max: Maximal oxygen consumption
rpm: Revolutions per minute
s: Second
min: Minute
h: Hour
Fe: Serum iron
WBC: White blood cells
RBC: *Red blood cells*
HGB: Haemoglobin
HCT: Haematocrit
MCV: *Corpuscular volume*
*MCH*: Corpuscular haemoglobin
MCHC: Corpuscular haemoglobin concentration
BMI: Body mass index
IL-6: Interleukin 6
TAC: Total antioxidant capacity
TAS: Total antioxidants status
TBARS: Thiobarbituric acid reactive substances
8-OHdG: Hydroxy-2′-deoxyguanosine
Nrf2: Nuclear factor erythroid 2-related factor 2
ARE: Antioxidative response
RNA: Ribonucleic acid
DNA: Deoxyribonucleic acid
LDL: Low-density lipoprotein
kg: Kilogram
mmol: Millimole
Cd: Cadmium
MAP: Mitogen-activated protein
NF-κB: Nuclear factor kappa B
ROS: Reactive oxygen species
NO: Nitric oxide radical
eNOS: Endothelial nitric oxide synthase
iNOS: *Inducible nitric oxide synthase*
PGC-1α: Peroxisome proliferator-activated receptor gamma coactivator 1-alpha
mtTFA: Mitochondrial transcription factor A
LOOH: Lipid hydroperoxides
*EIMD*: Exercise-induced muscle damage
PGE2: Prostaglandin E2
TNF α: *Tumour necrosis factor α*
et al.: And others
KB: Bioethics committee

## References

[1] Hurst SM, Lyall KA, Hurst RD, Stevenson LM (2009). Exercise-induced elevation in plasma oxidative generating capability augments the temporal inflammatory response stimulated by lipopolysaccharide. Eur J Appl Physiol.

[2] Woods JA, Pence BD (2015). Physical activity, exercise, and the immune system: three lines of research that have driven the field. Kinesiol Rev.

[3] Sloth M, Sloth D, Overgaard K, Dalgas U (2013). Effects of sprint interval training on VO2max and aerobic exercise performance: A systematic review and meta-analysis. Scand J Med Sci Sports.

[4] Rani V, Deep G, Singh RK, Palle K, Yadav UC (2016). Oxidative stress and metabolic disorders: pathogenesis and therapeutic strategies. Life Sci.

[5] Husain N, Mahmood R (2017). Hexavalent chromium induces reactive oxygen species and impairs the antioxidant power of human erythrocytes and lymphocytes: decreased metal reducing and free radical quenching ability of the cells. Toxicol Ind Health.

[6] Tangudu NK, Alan B, Vinchi F, Wörle K, Lai D, Vettorazzi S, Leopold K, Vujić SM (2018). Scavenging reactive oxygen species production normalizes Ferroportin expression and ameliorates cellular and systemic Iron Disbalances in hemolytic mouse model. Antioxid Redox Signal.

[7] Valko M, Morris H, Cronin MTD (2005). Metals, toxicity and oxidative stress. Curr Med Chem.

[8] Michailidis Y, Jamurtas AZ, Nikolaidis MG, Fatouros IG, Koutedakis Y, Papassotiriou I, Kouretas D (2007). Sampling time is crucial for measurement of aerobic exercise-induced oxidative stress. MSSE..

[9] Nikolaidis MG, Paschalis V, Giakas G, Fatouros IG, Koutedakis YI, Kouretas D, Jamurtas AZ (2007). Decreased blood oxidative stress after repeated muscle-damaging exercise. Med Sci Sports Exerc.

[10] Bresgen N, Eckl P (2018). Oxidative stress and cell death: the role of iron. Free Radic Biol Med.

[11] Baltopoulos P (2009). Exercise induced modulation of immune system functional capacity. Biol Exerc.

[12] Walsh NP, Oliver SJ (2016). Exercise, immune function and respiratory infection: an update on the influence of training and environmental stress. Immunol Cell Biol.

[13] Ward R, Crichton R, Taylor D, Corte L, Srai S, Dexter D (2011). Iron and the immune system. J Neural Transm.

[14] Gleeson M, Pyne DB (2016). Respiratory inflammation and infections in high-performance athletes. Immunol Cell Biol.

[15] Sikora J, Markowicz M. Biologically active compounds of fruit Aronia melanocarpa (Aronia melanocarpa Elliot). Oxidative Med Cell Longev. 2014:739721. 10.1155/2014/739721 PMC4094718 25050143

[16] Sueiro L, Yousef GG, Seigler D, De Mejia EG, Grace MH, Lila MA (2006). Chemopreventive potential flavonoid extracts from plantation-bred and wild Aronia melanocarpa (black chokeberry) fruits. J Food Sci.

[17] Borowska S, Brzóska MM (2016). Chokeberries (Aronia melanocarpa) and their products as a possible means for the prevention and treatment of noncommunicable diseases and unfavorable health effects due to exposure to xenobiotics. Compr Rev Food Sci Food Saf.

[18] Rodríguez WM, Esatbeyoglu T, Winterhalter P (2019). Phenolic composition, radical scavenging activity and an approach for authentication of Aronia melanocarpa berries, juice, and pomace. J Food Sci.

[19] Gajic D, Saksida T, Koprivica I, Vujicic M, Despotovic S, Savikin K, Jankovic T, Stojanovic I (2020). Chokeberry (Aronia melanocarpa) fruit extract modulates immune response in vivo and in vitro. J Funct Foods.

[20] Ho GTT, Bräunlich M, Austarheim I, Wangensteen H, Malterud KE, Slimestad R, Barsett H (2014). Immunomodulating Activity of *Aronia melanocarpa* Polyphenols. Int J Mol Sci.

[21] Jakovljevic V, Milic P, Bradic J, Jeremic J, Zivkovic V, Srejovic I, Nikolic Turnic T, Milosavljevic I, Jeremic N, Bolevich S, Labudovic Borovic M, Mitrovic M, Vucic V (2018). Standardized *Aronia melanocarpa* Extract as Novel Supplement against Metabolic Syndrome: A Rat Model. Int J Mol Sci.

[22] Cook MD, Willems MET (2019). Dietary anthocyanins: a review of the exercise performance effects and related physiological responses. Int J Sport Nutr Exercise Metab.

[23] Pilaczynska-Szczesniak L, Skarpanska-Steinborn A, Deskur E, Basta P (2005). M Horoszkiewicz-Hassan M. the influence of chokeberry juice supplementation on the reduction of oxidative stress resulting from an incremental rowing ergometer exercise. Int J Sport Nutr Exerc Metab.

[24] McLeay Y, Stannard S, Houltham S, Starck C (2017). Dietary thiols in exercise: oxidative stress defence, exercise performance, and adaptation. J Int Soc Sports Nutr.

[25] McLeay Y, Stannard S, Houltham S, Starck C. Dietary thiols in exercise: oxidative stress defence, exercise performance, and adaptation. J Int SocSports Nutr. 2017;14:12-19. 10.1186/s12970-017-0168-9 PMC5408473 28465675

[26] Hawkins J, Hires C, Baker C, Keenan L, Bush M (2020). Daily supplementation with *Aronia melanocarpa* (chokeberry) reduces blood pressure and cholesterol: a meta analysis of controlled clinical trials. J Diet Suppl.

[27] Oszmiansk J, Sapis JC (1988). Anthocyanins in fruits of Aronia melanocarpa (chokeberry). J Food Sci.

[28] Leger L, Mercier D, Gadoury C, Lambert J (1988). The multistage 20 metre shuttle run test for aerobic fitness. J Sports Sci.

[29] Léger LA, Lambert J (1982). A maximal multistage 20-meter shuttle run test to predict V˙O2max. Eur J Appl Physiol.

[30] Peeling P, Dawson B, Goodman C, Landers G, Wiegerinck ET, SwinkelsDW, Trinder D. effects of exercise on hepcidin response and iron metabolism duringrecovery. Int J Sport Nutr Exerc Metab. 2009; 19(6):583–597. doi:10.1123/ijsnem.19.6.583. PMID: 20175428. 10.1123/ijsnem.19.6.583 20175428

[31] Cipryan L (2017). IL-6, antioxidant capacity andMuscle damage markers following high-intensity interval training protocols. J Hum Kinet.

[32] Petrovic S, Arsic A, Glibetic M, Cikiriz N, Jakovljevic V, Vucic V (2016). The effects of polyphenol-rich chokeberry juice on fatty acid profiles and lipid peroxidation of active handball players: results from a randomized, double-blind, placebo-controlled study. Can J Physiol Pharmacol.

[33] R Core Team (2013). R: a language and environment for statistical computing.

[34] De Rosario-Martinez H. Phia: post-hoc interaction analysis. R Pack Vers. 2015:2–1 https://CRAN.R-project.org/package=phia.

[35] Bates D, Mächler M, Bolker B, Walker S (2015). Fitting Linear Mixed-Effects Models Using *lme4*. J Stat Softw.

[36] Kuznetsova A, Brockhoff PB, Christensen RHB (2017). Lmertest Package: Tests in Linear Mixed Effects Models. J Stat Softw.

[37] Smith LL (2000). Cytokine hypothesis of overtraining: a physiological adaptation to excessive stress?. Med Sci Sports Exerc.

[38] Oszmiański J, Wojdylo A (2005). Aronia melanocarpa phenolics and their antioxidant activity. Eur Food Res Technol.

[39] Malinowska J, Oleszek W, Stochmal A, Olas B (2013). The polyphenol-rich extracts from black chokeberry and grape seeds impair changes in the platelet adhesion and aggregation induced by a model of hyperhomocysteinemia. Eur J Nutr.

[40] Tolić MT, Landeka Jurčević I, Panjkota Krbavčić I, Marković K, Vahčić N (2015). Phenolic content, antioxidant capacity and quality of chokeberry (Aronia melanocarpa) products. Food Technol Biotechnol.

[41] Van Acker SA, Tromp MN, Haenen GR, van der Vijgh WJ, Bast A (1995). Flavonoids as scavengers of nitric oxide radical. Biochem Biophys Res Commun.

[42] Kokotkiewicz A, Jaremicz Z, Luczkiewicz M (2010). Aronia plants: a review of traditional use, biological activities, and perspectives for modern medicine. J Med Food.

[43] Kim B, Ku CS, Pham TX, Park Y, Martin DA, Xie L, Taheri R, Lee J, Bolling BW (2013). Aronia melanocarpa (chokeberry) polyphenol-rich extract improves antioxidant function and reduces total plasma cholesterol in apolipoprotein E knockout mice. Nutr Res.

[44] Nishiie-Yano R, Hirayama S, Tamura M, Kanemochi T, Ueno T, Hirayama A, Hori A, Ai T, Hirose N, Miida T (2020). Hemolysis is responsible for elevation of serum Iron concentration after regular exercises in judo athletes. Biol Trace Elem Res.

[45] Tedesco I, Moccia S, Volpe S, Alfieri G, Strollo D, Bilotto S, Spagnuolo C, Di Renzo M, Aquino RP, Russo GL (2016). Red wine activates plasma membrane redox system in human erythrocytes. Free Radic Res.

[46] Kapci B, Neradová E, Čížková H, Voldřich M, Rajchl A, Capanoglu E (2013). Investigating the antioxidant potential of chokeberry (Aronia melanocarpa) products. J Food Nutr Res.

[47] Cikiriz N, Milosavljevic I, Jakovljevic B, Bolevich S, Jeremic J, Nikolic Turnic T, Mitrovic M, Srejovic I, Bolevich S, Jakovljevic V (2020). The influences of chokeberry extract supplementation on redox status and body composition in handball players during competition phase. Can J Physiol Pharmacol.

[48] García-Flores LA, Medina S, Cejuela-Anta R, Martínez-Sanz JM, Abellán Á, Genieser H-G, Ferreres F, Gil-Izquierdo Á (2016). DNA catabolites in triathletes: effects of supplementation with an aronia-citrus juice (polyphenols-rich juice). Food Funct.

[49] Zügel M, Treff G, Steinacker JM, Mayer B, Winkert K, Schumann U (2020). Increased hepcidin levels during a period of high training load do not alter iron status in male elite junior rowers. Front Physiol.

[50] Villaño D, Vilaplana C, Medina S, Algaba-Chueca F, Cejuela-Anta R, Martínez-Sanz JM, Ferreres F, Gil-Izquierdo A (2016). Relationship between the ingestion of a polyphenol-rich drink, hepcidin hormone, and long-term training. Molecules..

[51] Skarpańska-Stejnborn A, Basta P, Sadowska J, Pilaczyńska-Szcześniak L (2014). Effect of supplementation with chokeberry juice on the inflammatory status and markers of iron metabolism in rowers. J Int Soc Sports Nutr.

[52] Bonarska-Kujawa D, Pruchnik H, Kleszczyńska H (2012). Interaction of selected anthocyanins with erythrocytes and liposome membranes. Cell Mol Biol Lett.

[53] Hider RC, Liu ZD, Khodr HH (2001). Metal chelation of polyphenols. Methods Enzymol.

[54] Seeram NP, Nair MG (2002). Inhibition of lipid peroxidation and structure–activity-related studies of the dietary constituents anthocyanins, anthocyanidins, and catechins. J Agric Food Chem.

[55] Qin B, Anderson RA (2012). An extract of chokeberry attenuates weight gain and modulates insulin, adipogenic and inflammatory signalling pathways in epididymal adipose tissue of rats fed a fructose-rich diet. Br J Nutr.

[56] Ohgami K, Ilieva I, Shiratori K, Koyama Y, Jin XH, Yoshida K, Kase S, Kitaichi N, Suzuki Y, Tanaka T, Ohno S (2005). Anti-inflammatory effects of aronia extract on rat endotoxin-induced uveitis. Invest Ophthalmol Vis Sci.

[57] Koivisto AE, Olsen T, Paur I, Paulsen G, Bastani NE, Garthe I, Raastad T, Matthews J, Blomhoff R, Bøhn SK (2019). Effects of antioxidant-rich foods on altitude-induced oxidative stress and inflammation in elite endurance athletes: a randomized controlled trial. PLoS One.

